# Automatic continuous on-line monitoring of salicylic acid and acetylsalicylic acid in pharmaceuticals

**DOI:** 10.1155/S1463924690000335

**Published:** 1990

**Authors:** J. M. López Fernández, M. D. Luque de Castro, M. Valcárcel

**Affiliations:** 1 Department of Analytical Chemistry ETSIA Córdoba 14004 Spain; 2 Faculty of Sciences University of Córdoba Córdoba 14004 Spain

## Abstract

An asymmetrical FIA merging-zones manifold based on the dual injection of two sample microvolumes was developed for the simultaneous determination of salicylic acid and acetylsalicylic acid in pharmaceutical preparations at a sampling frequency of 30/h. The complex formed between the Fe(III) reagent continuously introduced in the system and salicylic acid was monitored photometrically at 520 nm. One of the sample plugs was prehydrolysed on injection into an NaOH stream and was circulated through a longer channel than the other plug. This yielded two FIA peaks corresponding to salicylic acid and the overall content, respectively. The proposed manifold was successfully used to control the dissolution test of a pharmaceutical preparation.


Journal of Automatic Chemistry, Vol. 12, No. 6 (November-December 1990), pp. 263-266

### Automatic continuous on-line monitoring of salicylic acid and acetylsalicylic acid in pharmaceuticals

**J. M. López Fernández,† M. D. Luque de Castro‡ and M. Valcárcel‡**

*† Department of Analytical Chemistry, ETSIA, and ‡ Faculty of Sciences, University of Córdoba, 14004 Córdoba, Spain*

*An asymmetrical FIA merging-zones manifold based on the dual injection of two sample microvolumes was developed for the simultaneous determination of salicylic acid and acetylsalicylic acid in pharmaceutical preparations at a sampling frequency of 30/h. The complex formed between the Fe(III) reagent continuously introduced in the system and salicylic acid was monitored photometrically at 520 nm. One of the sample plugs was prehydrolysed on injection into an NaOH stream and was circulated through a longer channel than the other plug. This yielded two FIA peaks corresponding to salicylic acid and the overall content, respectively. The proposed manifold was successfully used to control the dissolution test of a pharmaceutical preparation.*

### Introduction

Flow Injection Analysis (FIA) is by now a solidly established analytical methodology [1-3], as reflected by the large number of papers (over 2900) published on the topic and by the variety of FIA analysers commercially available. FIA is entering a new age in which it will address real analytical problems. Thus, FIA can now be regarded as a major alternative in the automation of analytical processes; this is one of the main goals of contemporary analytical chemistry according to the US National Institute for Science and Technology (NIST) [4].

The growing significance of the role played by analytical chemistry in process control is now widely acknowledged [5,6]. The ready implementation of on-line process monitoring by using FIA analysers [7] allows for a fairly high degree of automation, as compared with the at-line and off-line alternatives, and facilitates calibration with respect to in-line and invasive methodologies [8]. The inherent advantages of FIA as an alternative to conventional approaches to monitoring pharmaceutical dissolution tests [9] have been discussed in depth elsewhere [10]. A significant advantage is the possibility of determining not only the active component in the sample, but also its degradation products.

In addition, FIA allows the ready implementation of speciation studies [11], such as the determination of free and combined analytes in various matrices by splitting the injected sample plugs into two subplugs, one of which is usually subjected to a previous – normally hydrolysis – reaction. The two channels through which the subplugs are circulated vary in length, so the latter reach the detection point at a different time. The result is two FIA peaks proportional to the free and overall analyte concentration. An example of this approach is the determination of free and combined sulphur dioxide in wines by double stopped-flow injection analysis [12]. This paper reports the determination of salicylic (SA) and acetylsalicylic (ASA) acid in pharmaceuticals by using an FIA manifold. The wellknown colour-forming complexation reaction between Fe(III) and salicylic acid was monitored photometrically at 520 nm. Once optimized, the manifold was applied to the dissolution testing of aspirin tablets. Although SA had been previously determined alone by FIA [13] and in mixtures with ASA by HPLC [14], their simultaneous assay by the latter technique, which is much more straightforward and convenient, has not been addressed to date.

### Experimental

*Reagents*

(1) Aqueous solution of $Fe(NO_3)_3 \cdot 9H_2O$ of pH 2·4 containing 1·0 g/l Fe(III).

(2) Aqueous 0·45 M HCl and 0·50 M NaOH solutions.

(3) Aqueous 1·0 g/l salicylic acid and 2·0 g/l acetylsalicylic acid solutions from which more dilute solutions were prepared as required.

All reagents used were of analytical grade.

*Apparatus*

A Pye-Unicam SP-500 single-beam spectrophotometer furnished with a Hellma 178·12 QS flow-cell and connected to a Radiometer recorder was used. A four-channel Gilson Minipuls-2 peristaltic pump, provided with a rate selector and a custom-made injection valve consisting of two Rheodyne 5041 single injection valves, was also used.

*General procedure*

The manifold used is shown in figure 1. The sample, an aqueous solution containing SA, ASA or both, was aspirated by means of the peristaltic pump and two microvolumes were simultaneously inserted into two HCl and NaOH streams, respectively, with the aid of the dual injection valve. The NaOH channel was somewhat longer and enacted the sample hydrolysis. By using an asymmetrical merging-zones assembly, a stream of the Fe(III) derivatizing reagent was merged with

0142-0453/90 $3.00 © 1990 Taylor & Francis Ltd.

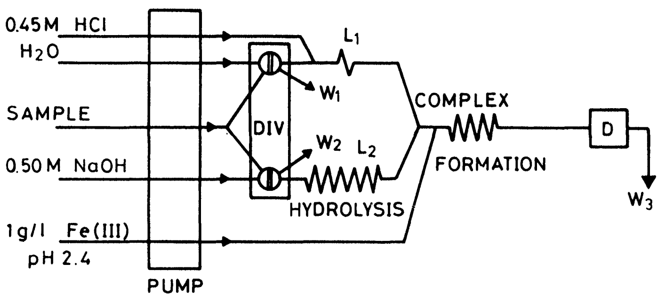

*Figure 1. FIA manifold for the simultaneous determination of salicylic and acetylsalicylic acid. DIV: dual injection valve; D: detector; W: waste. For further details see text.*

common channel after the confluence point, so the sequential arrival of the two simultaneously injected plugs at the detector gave rise to two FIA peaks due to the absorbance of the complex formed between SA and Fe(III) in an acid medium. The first peak was due to SA alone, while the second corresponded to the contribution of the two acids.

*Dissolution test*

The manifold described above was modified for testing the aqueous dissolution of commercial aspirin tablets via the hydrolytic cleavage of acetylsalicylic acid (see figure 2). The closed FIA manifold used for this purpose involved injecting the sample from a reservoir and collecting the waste from the dual injection valve. Each sample injection yielded two profiles corresponding to the system releasing SA on hydrolysis, and to the overall, constant concentration of ASA. In this way, dissolution tests of commercial pharmaceutical preparations containing ASA can be readily controlled by monitoring its hydrolysis by FIA.

### Results and discussion

*Chemistry of the process*

*Hydrolysis reaction*

Aqueous SA solutions are stable in acid and alkaline media; ASA solutions, on the other hand, undergo hydrolysis according to:

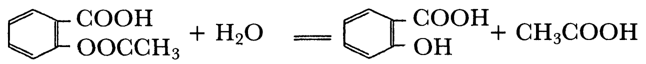

This process is accelerated in an alkaline medium. In this work the hydrolytic cleavage of ASA was exploited by having the second injection valve inserting the sample into a longer reactor, $L_2$, through which a 0·5 M NaOH solution was circulated to effect the hydrolysis; the AS yielded in the reaction was driven to the shorter reactor, $L_1$ (containing 0·45 M HCl), and later with the Fe(III) stream to obtain two sequential FIA peaks per injection of the dual valve. The kinetics of the hydrolysis reaction was reasonably fast and was completed by using 0·50 M NaOH at the longer reactor, $L_2$ (320 cm).

*Indicator reaction*

The monitored reaction was the formation of a blue complex ($\lambda_{max}$ = 520 nm) between SA and Fe(III) in an acid medium (pH 2·2–2·8). This required the use of an Fe(III) solution of a sufficiently high concentration to ensure adequate sensitivity, and a pH close to 2·4 to preserve an appropriate acidity after mixing at the NaOH-containing reactor in the hydrolysis stage. ASA yields no coloured product with Fe(III).

*Optimization of the system*

*Chemical variables*

The chemical variables affecting the system performance were the NaOH concentration used in the hydrolysis of ASA; the Fe(III) concentration, which determined the sensitivity level achieved, and the HCl concentration required to restore the medium acidity required for the indicator reaction (formation of the FeSA complex).

*FIA variables*

The FIA variables optimized in this case were the sample volumes injected via the loops of the dual valve, the flow-rate and the reactor dimensions.

Table 1 lists the ranges over which these variables were assayed and the optimum values found.

*Figures of merit*

*Determination of SA*

Under the optimum working conditions, SA standards of concentrations between 100 and 1000 mg/l were injected into the proposed manifold. The calibration graph run was linear over the range 100–600 mg/l SA. The equation of the calibration line obtained from the first peak was A = 0·163 + 1·1 × $10^{-3}$ [AS], with a regression coefficient larger than 0·99. The reproducibility (expressed as rsd) found for 11 samples of SA, with a concentration of 400 mg/l injected in triplicate, was ±0·36%, and the sample throughput was 30 samples/h.

*Determination of ASA*

Under the optimum working conditions, ASA standards of concentrations between 300 and 2000 mg/l were injected into the proposed manifold. The calibration graph run was linear over the range 300–1800 mg/l ASA. The equation of the calibration line obtained from the second peak was A = −0·163 + 2·13 × $10^{-3}$ [AS], with a regression coefficient larger than 0·99. The rsd, calculated as in the case for an ASA concentration of 600 mg/l, was ±0·48%, and the sample throughput was 30 samples/h.

*Determination of mixtures*

To test the applicability of the proposed method to SA-ASA mixtures, samples were diluted so that their concentrations were within the above linear ranges. The concentrations, recoveries and errors found (table 2) testify to the highly encouraging performance of the method.

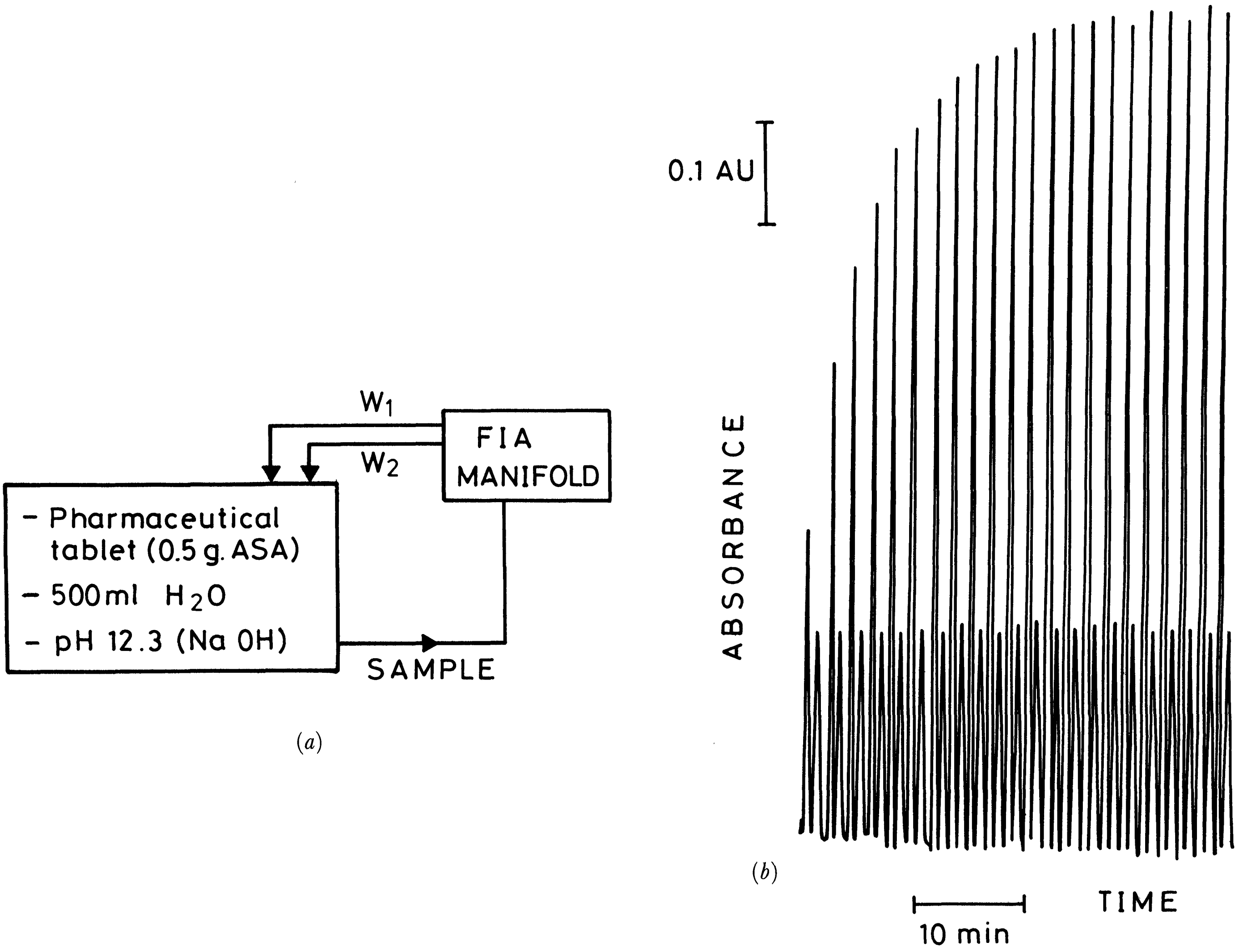

*Figure 2. Implementation of an automatic assembly for the monitoring of the dissolution of an aspirin tablet (Bayer). (a) Evolving system connected on-line to the FIA manifold in figure 1. (b) FIA signals obtained.*

*Table 1. Assayed ranges and optimum values of experimental variables.*

| | [HCl] (mol/l) | [NaOH] (mol/l) | [Fe(III)] (μg/ml) | Sample volume $V_1$ (μl) | Sample volume $V_2$ (μl) | Flow rate (ml/min) |
|---|---|---|---|---|---|---|
| Assayed range | 0·1–1·0 | 0·1–1·0 | 100–200 | 30–300 | 30–300 | 0·5–5·0 |
| Optimum value | 0·45 | 0·50 | 1000 | 100 | 58 | 1·5 |

Reactor lengths: $I_1$ = 20 cm; $L_2$ = 320 cm.

*Table 2. Determination of SA–ASA mixtures.*

| Sample | [SA] Present (μg/ml) | [SA] Found (μg/ml) | [SA] Error (%) | [ASA] Present (μg/ml) | [ASA] Found (μg/ml) | [ASA] Error (%) |
|---|---|---|---|---|---|---|
| 1 | 800 | 770 | −3·7 | 1200 | 1202 | +0·1 |
| 2 | 200 | 198 | −1·0 | 600 | 666 | +11·0 |
| 3 | 200 | 209 | +4·5 | 1200 | 1235 | +2·9 |
| 4 | 600 | 644 | +7·3 | 1800 | 1638 | −9·0 |
| 5 | 200 | 219 | +9·5 | 1800 | 1763 | −2·0 |
| 6 | 600 | 616 | +2·6 | 600 | 629 | +4·8 |

*Continuous monitoring of dissolution tests*

One of the major current rends in the use of FIA is for controlling natural, simulated and industrial processes. A manifold has been adapted to monitor the hydrolytic cleavage of ASA in commercial aspirin tablets. The first peak obtained was due to the system evolution, while the second resulted from the overall, constant concentration of ASA in the system.

### Conclusions

The results obtained in this work prove the suitability of FIA for simultaneous determinations of pharmacological interest and in controlling simulated processes (dissolution tests), particularly when the active component of the pharmaceutical cannot be sensed directly.

### References

1. Valcarcel, M. and Luque de Castro, M. D., *Flow Injection Analysis. Principles and Applications* (Ellis Horwood, Chichester, 1987).
2. Ružička, J. and Hansen, E. H., *Flow Injection Analysis*, 2nd edn (Wiley, New York, 1988).
3. Karlberg, B. and Pacey, G., *Flow Injection Analysis. Principles and Applications* (Elsevier, Amsterdam, 1989).
4. Kingston, H. M., *Analytical Chemistry,* **61** (1989), 1381 A.
5. Callis, J. B., Ilman, D. L. and Kowalsaki, B. R., *Analytical Chemistry,* **59** (1987), 626A.
6. Van der Linden, W. E., *Analytica Chimica,* **61** (1989), 1381A.
7. Luque de Castro, M. D., *Talanta,* **36** (1989), 591.
8. Luque de Castro, M. D. and Valcarcel, M. D., *Journal of Automatic Chemistry* (in press).
9. United States Pharmacopoeia, 20th rev. (US Pharmacopeial Convention, Rockville, Maryland, 1980), 57.
10. Luque de Castro, M. D. and Valcarcel, M., *Journal of Pharmaceutical and Biomedical Analysis* (in press).
11. Luque de Castro, M. D., *Talanta,* **33** (1985), 45.
12. Lazaro, F., Luque de Castro, M. D. and Valcarcel, M. D., *Analytical Chemistry,* **59** (1987), 950.
13. Chang, Q. and Meyerhoff, M. E., *Analytica Chimica Acta,* **186** (1986), 81.
14. Fogel, J., Epstein, P. and Chen, P. J., *Journal of Chromatography,* **317** (1984), 507.

